# Attention and sentiment of Chinese public toward rural landscape based on Sina Weibo

**DOI:** 10.1038/s41598-024-64527-1

**Published:** 2024-06-14

**Authors:** Jinji Zhang, Guanghu Jin, Yang Liu, Xiyue Xue

**Affiliations:** 1 Yanbian University, College of Agriculture, Yanji, 133002 China; 2 Yanbian University, College of Engineering, Yanji, 133002 China

**Keywords:** Environmental impact, Computer science

## Abstract

Rural landscapes, as products of the interaction between humans and nature, not only reflect the history and culture of rural areas but also symbolize economic and social progress. This study proposes a deep learning-based model for Weibo data analysis aimed at exploring the development direction of rural landscapes from the perspective of the Chinese public. The research reveals that the Chinese public’s attention to rural landscapes has significantly increased with the evolution of government governance concepts. Most people express a high level of satisfaction and happiness with the existing rural landscapes, while a minority harbor negative emotions towards unreasonable new rural construction. Through the analysis of public opinion regarding rural landscapes, this study will assist decision-makers in understanding the mechanisms of public discourse on social media. It will also aid relevant scholars and designers in providing targeted solutions, which hold significant importance for policy formulation and the exploration of specific development patterns.

## Introduction

Traditional economic growth models often overlook the sustainability of the environment and society, leading to various environmental challenges such as climate change, biodiversity loss, resource depletion, and land and environmental degradation^1^. In this context, the international community has agreed upon the necessity of ecological civilization construction to achieve sustainable development. Landscape construction, which creates spaces in harmony with nature, is an important component of ecological civilization construction, thereby promoting a balance between socioeconomic development and environmental protection^2^. Examples include increased green coverage to reduce the urban heat island effect^3^, “green infrastructure” projects for flood mitigation^4^, ecological corridors for wildlife habitats and migration^5^, and plant cultivation for enhanced carbon sequestration^6^. All these efforts contribute to the Sustainable Development Goals, focusing on urban community sustainability and growth. Rural landscapes, possessing abundant natural resources and scope for transformation, have become the focus in landscape architecture, especially with the rise of rural tourism^7^. Various rural landscape evaluation standards (e.g., SITES in the United States, BREEAM in the United Kingdom, DGNB in Germany, and China’s national standard GB/T37072-2018 “Assessment for Beautiful Rural Construction”) have promoted rural landscape development. Nevertheless, despite promotional endeavors, the consumer market’s response to rural landscapes remains underwhelming due to excessive government intervention and insufficient market participation^8^. The 19th National Congress of the Communist Party of China proposed the goal of establishing an environmental governance pattern led by the government with broad participation from social organizations and the public. Simultaneously, documents such as the “Opinions on Strengthening the Improvement of Rural Living Environment”, the “Rural Revitalization Strategy (2018–2022),” and the “Regulations on Rural Collective Economic Organizations” emphasize the role of the public in the decision making process and highlight the significance of their involvement.

Rural landscapes encompass various elements within rural areas, including natural scenery, manmade structures, farmland, water bodies, and vegetation. It also involves the combination and interaction among these elements^9^. Research on rural landscapes in China has achieved fruitful results in terms of research perspectives, theoretical foundations, and application methods. Simultaneously, studies on the mechanisms of public attention to rural landscapes have gradually increased and deepened. Zhu et al.^10^, combining public preferences with professional theories and through the evaluation of rural landscape photographs, have revealed the positive correlation between the visual quality of rural landscapes and elements such as terrain, forests, streams, and traditional buildings. Cheng et al.^11^ start from the concept of public participation, analyzing the existing issues in villagers’ participation in the creation of microlandscapes. Su et al.^12^, employing survey interviews, the Delphi method, hierarchical analysis, and case study methods, have developed an evaluation index system for public participation in rural landscape construction. Peng et al.^13^ have dissected the guidance and shortcomings of public participation in rural landscape planning practice from three aspects: participation entities, stages and methods of participation, and the results of participation. Zhang et al.^14^ conducted a content analysis of photos posted by the public on social media, summarizing the public’s preferences for rural landscapes. However, existing research still has shortcomings. First, the extent of public attention to rural landscapes is inadequately addressed in existing studies. Second, current research is primarily static and lacks socioeconomic dynamism. Third, existing research on public perceptions of rural landscapes mainly emphasize participant satisfaction, whereas the emotional orientation and focal points of public attention remain unexplored.

As a mainstream social media platform in China, Sina Weibo has 586 million monthly active users and remarkable user engagement, becoming a crucial channel for netizens to communicate and gather information^15^. The reliability of Weibo as a data source has been thoroughly investigated by Zhang et al.^16^. Additionally, research by Jiang et al.^17^ has demonstrated that, provided there is a sufficient volume of data, complete user information, and ample textual content, and the data is processed using scientific core technologies, it satisfies the prerequisites for being employed as research data. In this paper, we utilize the Sina Weibo platform, coupled with web scraping techniques and various text mining methods, to investigate the dynamic emotional orientation and shifting focus of the Chinese public towards rural landscapes. Our goal is to offer recommendations for the future development of rural landscapes and provide new research perspectives and data foundations for similar studies. By analyzing user behaviors on Weibo, we first investigate the Chinese public’s attention to rural landscapes across temporal and spatial dimensions . Secondly, we explore the emotional orientations of the Chinese public towards rural landscapes, specifically focusing on positive and negative emotions. Next, we perform a thematic analysis of the content of posts reflecting these emotional orientations. Finally, we analyze the development trends of different themes .

Our main contributions in this work are summarized as follows:

- This research systematically examines how the Chinese public perceives and responds to changes in rural landscapes from the perspective of public opinion analysis, offering valuable insights into the developmental trajectories of rural landscapes in China.
- This research reveals the Chinese public’s multifaceted concerns, emotional responses, key focus areas, and future projections regarding rural landscapes, providing a new data foundation for similar research.
- This research introduces a novel deep learning-based framework that integrates web scraping and BERT model analysis, significantly improving data collection and interpretation from Chinese social media platforms to advance understanding of rural landscapes within the contemporary socio-cultural context.

The remainder of this article is organized as follows. The “Method” section describes the methodology and technical framework. The “Result and analysis” section presents and examines the research findings. The “Discussion” section provides recommendations based on the research findings. The “Conclusion” section draws conclusions and provides a summary.

## Method

In this section, we will elaborate in detail on the methodology adopted in our study. This approach is divided into two core stages: (1) the data information extraction stage, implemented through web crawling technology; (2) the text analysis stage, conducted with text mining technology. Finally, we thoroughly describe the key components of our method.

###  Research framework and methodology

**Figure 1:**
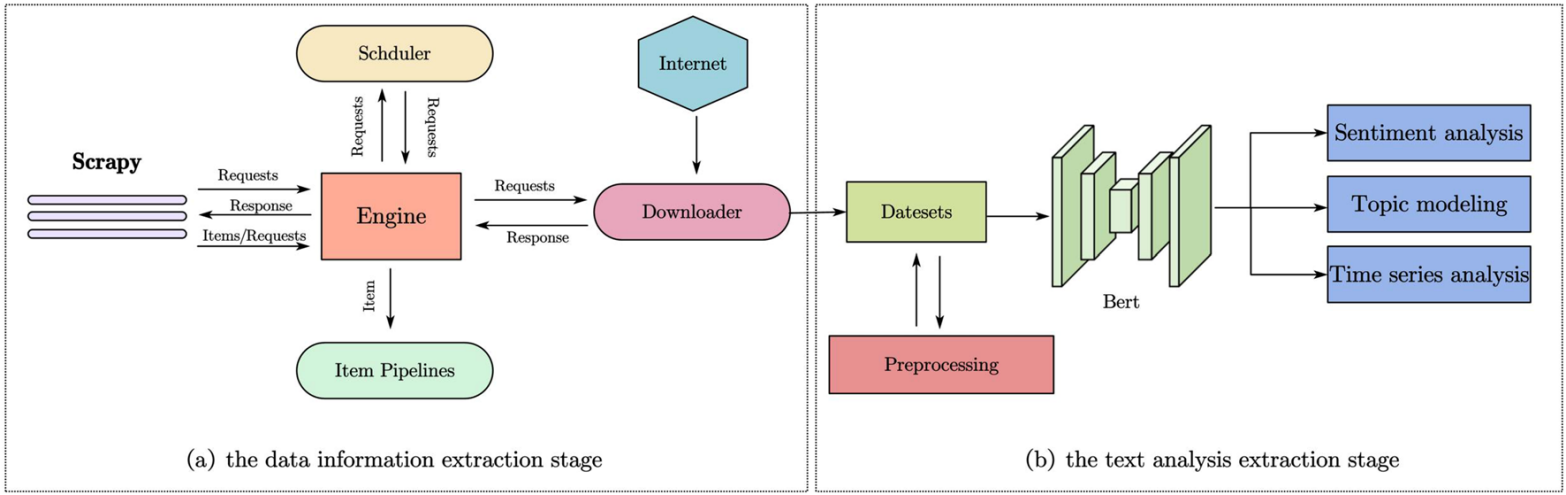
Research framework: (**a**) The data information extraction stage. (**b**) The text analysis stage.

This research is dedicated to systematically exploring the public’s attention, emotional tendencies, and thematic distribution towards rural landscapes, and further analyzing the profound significance and value of rural landscapes within the current socio-cultural context. Traditional data collection methods either rely on surveys and manual statistics or use archival research and group discussions to integrate information. However, these methods often consume significant human and material resources, and are prone to data biases, high non-response rates, and limitations related to geographical scope and scale. In response to these challenges, we propose a research framework based on deep learning. As shown in Fig. 1, this framework is divided into two stages: the data information extraction stage and the text analysis stage.

In the data information extraction stage, our framework employs the Scrapy Spider framework to design an efficient web crawler for collecting data from the Sina Weibo platform . This technique simulates the user login process to bypass traditional access restrictions, thereby accurately gathering information about rural landscapes, including article content, user interactions (such as comments and likes), timestamps, and geographical location information. Additionally, by implementing a carefully designed keyword filtering strategy, we effectively focus on data closely related to our research topic, creating a rich and accurate dataset for subsequent text analysis. The data collection method of this technology not only improves the accuracy and relevance of the data but also effectively overcomes the regional biases and limitations inherent in traditional manual data collection methods, ensuring the breadth and representativeness of the data. This allows us to delve deeper into the social and cultural value of rural landscapes.

In the Text Analysis Stage, after de-duplicating and normalizing the initially collected data based on similarity, we conducted an in-depth text analysis covering three main methods: sentiment analysis, topic modeling, and time series analysis. We first applied sentiment analysis to assess users’ emotional responses towards rural landscapes. For example, we analyzed specific Weibo posts, which described users’ positive reactions to the construction of new rural areas. Using a pre-trained BERT model, we accurately classified the emotions as positive or negative. Next, in topic modeling, using Bertopic technology, we identified the main topics related to ’rural landscape’ from thousands of Weibo posts . Lastly, in the time series analysis, we meticulously tracked and analyzed the developmental trend of the ’rural landscape’ theme over the past 5 years .

### The data information extraction stage

In the current era of information technology and big data, the prevalence and prosperity of the Internet have brought forth massive online information resources. To effectively extract and utilize these online resources, web crawling technology has emerged^18^. Web crawling is a technique that traverses the Internet by following hyperlinks, URLs, or web page indices^19^. It operates based on rules and algorithms, enabling automatic access and data extraction, thereby transforming raw data into valuable information.

In this study, to ensure the integrity and relevance of our research data, we focused on a significant period in Chinese rural development. In October 2017, the Chinese government introduced the Rural Revitalization Strategy ^20^, which significantly increased public engagement with rural landscapes. Consequently, we employed the keyword “rural landscape” to collect Weibo posts shared by the Chinese public from October 2017 through April 2023. This approach ensures that our dataset accurately reflects public opinion and engagement with rural landscapes during this critical period.

**Figure 2:**
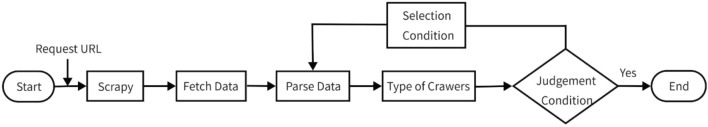
Scrapy Spide flowchart.

To collect this data, our methodology during the information extraction phase predominantly leverages the Scrapy Spider framework^21^, with the comprehensive flowchart illustrated in Fig. 2. The initial step entails a simulation of the login process, wherein the spider issues a POST request carrying username and password parameters to simulate user authentication on the Sina Weibo platform. This procedure acquires essential login state information such as cookies and authentication details, providing a foundational layer of authentication for subsequent operations.

During the data acquisition phase, our efforts were concentrated on extracting key pieces of information, including user nicknames, specific content of microblogs, posting times, geographical locations of the posts, and interaction data among users. This phase not only emphasized the importance of rich data content but also highlighted the spatial-temporal aspects of the data, facilitating a multifaceted analysis. Subsequently, the Scrapy framework transformed the harvested raw data into a structured format conducive to analysis. This transformation process involves sophisticated data parsing techniques to accurately decipher and structure the heterogeneous data types found within microblogs.

In the final stage of data collection, through meticulous verification processes, we ensured the integrity and accuracy of the data, exporting it without discrepancies for storage in a database. This rigorous validation process includes automated scripts to detect and rectify any inconsistencies, thereby guaranteeing the reliability of data for analysis and subsequent research endeavors.

Throughout the data extraction process, the “selection condition” and “judgment condition” decision nodes played a crucial role. The former is based on predefined selection criteria to determine whether to continue the data extraction process, while the latter concludes the process based on whether the data collection needs have been fully met or if predetermined termination conditions have been reached. When all these conditions are satisfied, the phase marked by the “end” node signifies the successful completion of the data extraction task. This rigorous and structured approach ensures a precise and efficient transition of data from the web to the database, laying a solid foundation for accurate data analysis and insightful research findings.

### The text analysis stage

**Figure 3:**
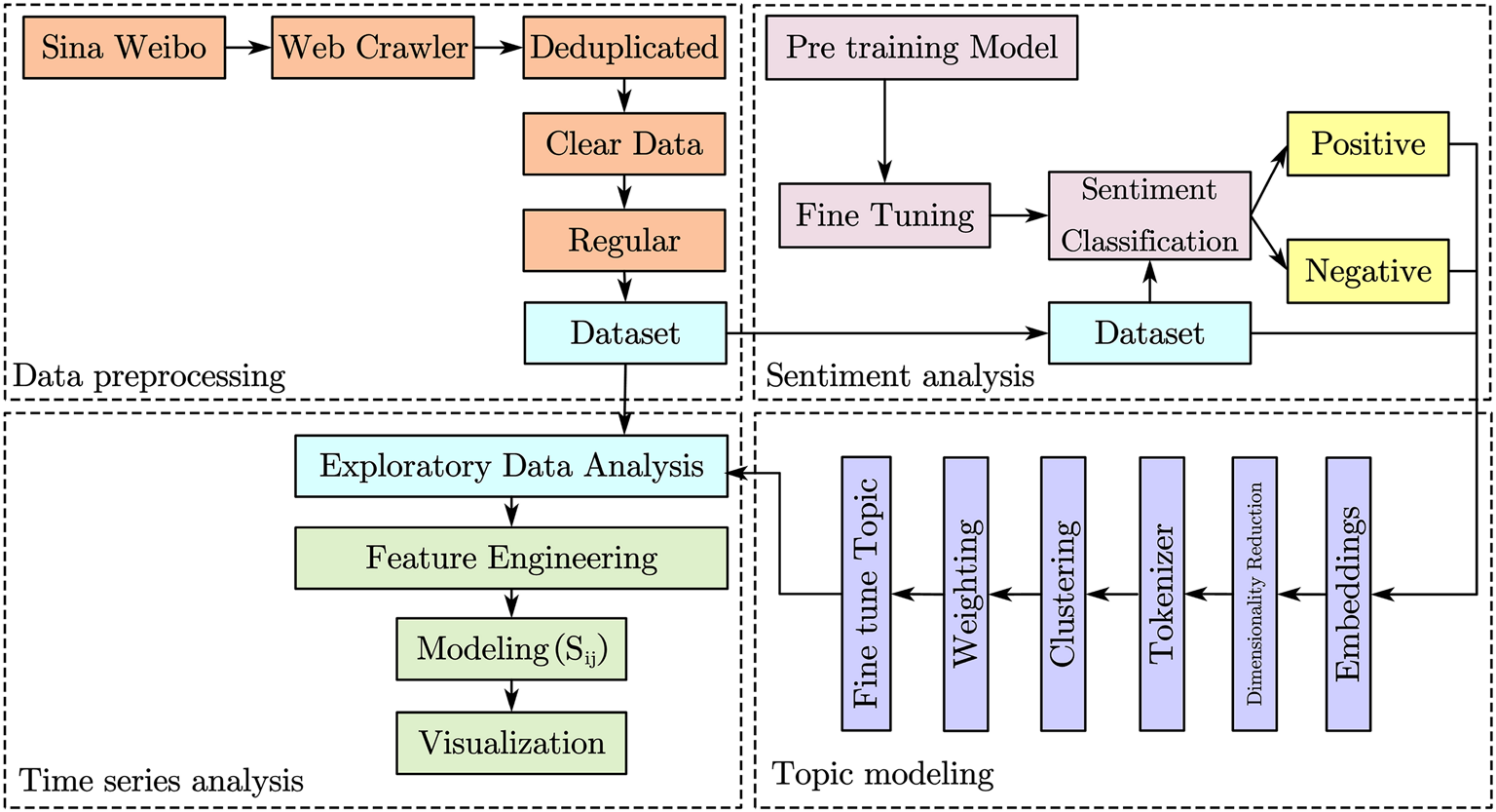
The text analysis stage framework.

In the text analysis stage, we leverage text mining techniques to extract valuable information and insights from the textual data obtained during the data information extraction stage. As shown in Fig. 3, this process begins with the collection of text data, followed by text preprocessing to clean up duplicate and incomplete textual data. Based on this preparatory work, we have established three main text analysis tasks: Sentiment Analysis, Topic Modeling, and Time Series Analysis, aiming to thoroughly mine and understand the insights hidden within the text data.

#### Text preprocessing

As shown in Fig. 3, the obtained text is subjected to similarity-based deduplication. To maximize deduplication, we use the Levenshtein distance^22^ to compute string similarity. By setting a threshold value, the rigor of the deduplication process is controlled.

$$\begin{aligned} \text {Similarity} = \frac{\text {MaximumLength} - \text {LevenshteinDistance}}{\text {MaximumLength}} \times 100, \end{aligned}$$

where $\text {Similarity}$ is the percentage similarity between two strings, $\text {MaximumLength}$ is the length of the longer string among the two being compared, and $\text {LevenshteinDistance}$ is the Levenshtein distance between the two strings, which measures the minimum number of single-character edits (insertions, deletions, or substitutions) required to change one word into the other.

Following deduplication, the next phase involves the cleansing of incomplete textual data. This step targets the removal of content deemed devoid of research value, such as advertisements, video text titles, and image text titles. This is achieved by identifying specific patterns or markers indicative of such content, utilizing a combination of keyword lists and structural analysis to accurately isolate and eliminate these data points. For example, consider a Weibo post that includes the text: “Watch our latest video on rural landscapes! [Video Link]”. By using keyword lists to identify the term “[Video Link]” and structural analysis to recognize “[Video Link]”, this content can be flagged and removed from the dataset.

Finally, the preprocessing stage addresses the elimination of noise and irrelevant information within the text through the application of regular expressions. This includes the removal of special characters, punctuation marks, HTML tags, URL links, images, and videos, which can obfuscate meaningful analysis. For instance, regular expressions such as /< [$^{\wedge }>$]*> /g to match HTML tags and / $\backslash$ bhttps?: $\backslash$ / $\backslash$ / $\backslash$ S+/gi to find URLs are utilized to clean the text data efficiently. By applying these preprocessing steps, the text data is refined, paving the way for more accurate and insightful analysis in subsequent phases of the research.

#### Sentiment analysis

Sentiment analysis aims to judge whether a text expresses positive or negative emotions by identifying and extracting subjective information within the text^23^. In this study, we constructed a sentiment analysis model using the “Fengshenbang-LM”^24^ pretrained model based on the BERT model.

Initially, model feature extraction function *f*(*x*) is applied to the preprocessed text data *T*, yielding the feature vector *V*, denoted as $V = f(T)$. Subsequently, this feature vector is fed into a classification layer. This layer maps the feature vector onto an output vector *Z*, whose dimensionality matches the number of emotional categories, with each element of the output vector corresponding to the logits of an emotional category. To transform these raw scores into class probabilities, the output from the classification layer is activated using the softmax function. For each category *i*, the softmax function is defined as follows:

$$\begin{aligned} Z_i & = W_i \cdot V + b_i,\\ P(class_i) & = \frac{e^{Z_i}}{\sum _{j} e^{Z_j}}, \end{aligned}$$

where $Z_i$ is the logit for class *i*, $W_i$ is the row vector in the weight matrix corresponding to class *i*, and $b_i$ is the bias term.Finally, the model makes a sentiment classification decision for a given input based on the class with the highest probability:

$$\begin{aligned} \text {Sentiment Class} = \arg \max _i P(class_i), \end{aligned}$$

where $P(class_i)$ represents the probability that the text belongs to sentimen class *i*. Ultimately, the model predicts the class that has the highest probability.

#### Topic modeling

The objective of topic modeling^25^ is to reveal the latent thematic structures within a collection of documents^26^. In this study, we utilize a BERT model, which has been fine-tuned for sentiment classification tasks, within the BERTopic framework^27^. As shown in Fig. 3, this framework combines transformer technology with the contextual Term Frequency-Inverse Document Frequency (c-TF-IDF) method^28^ to create dense thematic clusters, facilitating a more intuitive and understandable representation of the underlying data information. Initially, the Sentence-BERT framework is employed to enhance the embedding process, using pretrained language models to convert textual sentences and paragraphs into dense vector representations. Subsequently, to better categorize each thematic word into different topics, we apply the UMAP algorithm^29^ to reduce the dimensional representation of the aforementioned document embeddings to two or three dimensions. Then, the HDBSCAN^30^ algorithm is used to identify clusters of varying densities and designate certain data points as noise or outliers. Lastly, the c-TF-IDF method is utilized to generate keywords for each topic cluster. Through an iterative process, the c-TF-IDF representation of the least common topics is merged with the most similar topics, reducing the total number of topics to the desired amount.

#### Time series analysis

Time series analysis^31^ played a pivotal role in our study, especially in exploring and understanding the dynamics of changes in the content of Weibo posts over time. This analysis aims to identify and predict trends, periodic changes, and correlations between topics at different time points. By employing time series analysis, we were able to reveal the trends of specific keywords within Weibo posts over specified periods. To quantify the degree of correlation between topics and their changes over time, we introduced the theme strength coefficient $S_{ij}$ to represent the performance strength of theme *i* across a series of time points $T_i = \{ t_{i1}, t_{i2},..., t_{im} \}$ as follows:

$$\begin{aligned} S_{ij} = \lambda \cdot f(t_j, \text {topic}) + (1 - \lambda ) \cdot \sum _{k=1}^{j-1} \alpha _k \cdot S_{ik}, \end{aligned}$$

where $\lambda$ is a parameter between 0 and 1 that balances the importance of the current time point’s measurement against the historical strengths. is a function that provides a measurement for theme *i* at time point $t_{j}$, such as mention counts, user interactions. $\sum _{k=1}^{j-1} \alpha _k \cdot S_{ik}$ is the weighted sum of the theme’s strengths at all previous time points, indicating the cumulative effect of the past.

## Result and analysis

We collected Weibo posts and related information about rural landscapes from October 18, 2017 to April 30, 2023. A total of 81,745 pieces of data were collected, and after text preprocessing, 60,019 pieces of data remained.

### Analysis of temporal characteristics

**Figure 4:**
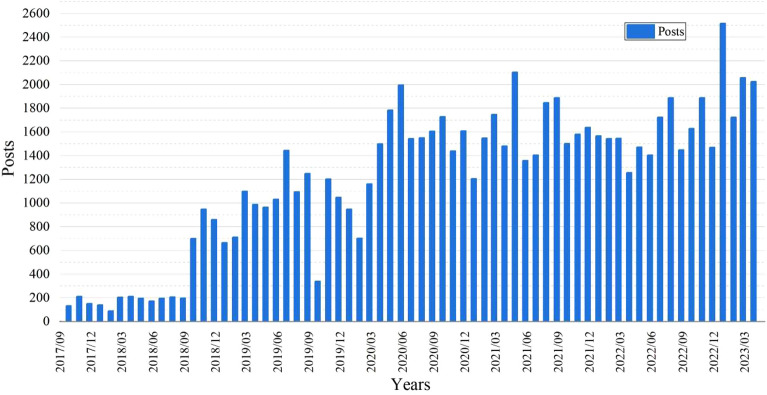
Timeline chart of posts.

A time series plot depicting the monthly posting volume based on the horizontal axis of time and the vertical axis of post count is shown in Fig. 4. Prior to September 2018, the post count was exceedingly low, reaching a monthly minimum of only 90 posts, signifying minimal public interest in the topic. Starting from October 2018, the topic gained sustained momentum with a notably elevated posting volume, indicating a substantial increase in public attention. In October 2019, a drop in post count was observed as public attention shifted to celebrating China’s 70th anniversary^32^. Subsequent months exhibited some growth in post count, albeit less pronounced than before, revealing a significant influence of the initial outbreak of the COVID-19 pandemic on posting behavior^33^. The year 2020, which marked the decisive push for building a moderately prosperous society in all respects, witnessed a peak in post count from June onward. A series of central government documents were issued during this period, promoting rural development and fueling public enthusiasm for exploration^34^. November 2020 saw another decline in post count, correlating with a resurgence of the pandemic. The post count reached its zenith in January 2023, reflecting heightened public interest in rural landscapes following the comprehensive lifting of pandemic restrictions. Overall, the post count related to rural landscapes exhibited a pattern of growth followed by fluctuation. Among the significant influencers were relevant policy documents, alongside major national events that garnered widespread attention^35^.

### Analysis of spatial characteristics

**Figure 5:**
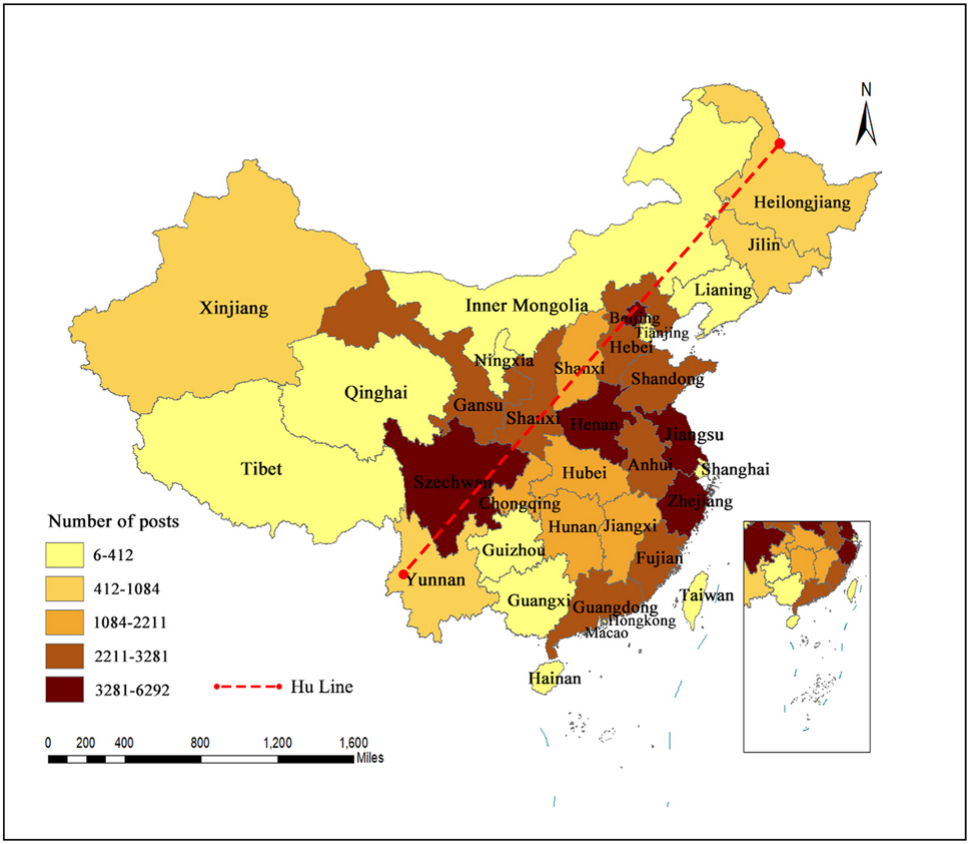
Regional distribution map (this map was created using ArcMap 10.7 software by ESRI. For more details, please visit: https://www.esri.com/).

In this study, we aggregated the IP addresses of individual Weibo posts by province to analyze the regional distribution patterns of postings related to rural landscapes , and the geographical distribution of Weibo posting volume is depicted in Fig. 5 (this map was created using ArcMap 10.7 software by ESRI. For more details, please visit: https://www.esri.com/). The color intensity in the Fig. 5 corresponds to the quantity of Weibo posts, with darker colors indicating higher quantities and lighter colors indicating lower quantities. In comparison with other provinces, Jiangsu, Zhejiang, and Henan Provinces exhibited the highest posting volumes, whereas Shandong, Hebei, and Shaanxi Provinces showed relatively higher posting volumes. Evidently, the geographical distribution of posting volume generally demonstrates a characteristic of higher activity in the southeastern coastal regions and lower activity in the northwestern coastal areas.

### Sentiment analysis

**Figure 6:**
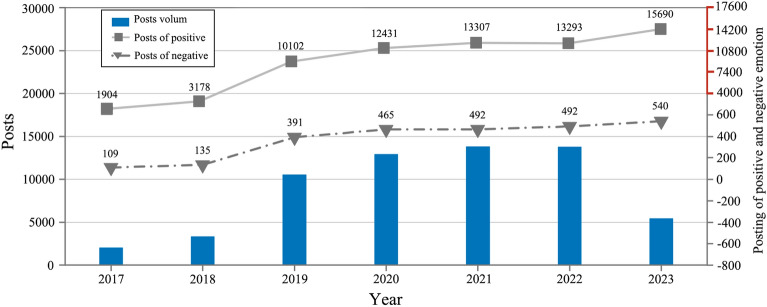
The number of positive and negative posts.

We utilized the Roberta pretrained model to finetune a dataset of 80,000 sentiment analysis samples, accomplishing the sentiment analysis task^36^. The range of emotion intensity values is between 0 and 1, where higher predicted values indicate stronger emotions. From a temporal perspective, we categorized the volume of posts expressing positive and negative emotions. The results are depicted in Fig. 6. Given the fact that posting volumes for 2017 and 2023 were only recorded for certain months, we conducted a proportional transformation of the data for these years.

The volume of posts expressing positive emotions demonstrates a year-on-year growth trend. Prior to 2018, the number of positive posts was minimal, indicating that after the introduction of the rural revitalization strategy, the Chinese public’s growing awareness of rural landscapes gradually emerged. In 2019, a sharp increase was observed in positive post data. During that year, the highest number of relevant policy documents were released, advocating for prioritized development of agriculture and rural areas, emphasizing rural resources as the foundation, and prioritizing the development of local industries and rural tourism. In the subsequent years, the number of posts consistently remained at a high level, indicating that these policies encouraged public attention toward rural landscapes. However, inconveniences during the pandemic led to relatively minor fluctuations in posting activity. Periodic decreases were also observed, with a drop of 68 posts in 2022 compared with the previous year. In 2023, the posting activity reached a peak, clearly illustrating heightened public enthusiasm for exploring rural landscapes in the post-pandemic era^37^.

The overall posting volume of negative emotions remains consistent with the annual posting volume, and compared to previous years, the number of negative posts has increased. Evidently, as the posting volume continues to grow, the prevalence of negative emotions has also increased. Thus, alongside the ongoing rural landscape development, the public has also expressed a certain degree of concern.

### Topic analysis

Topic modeling is often used to explore the thematic structure of large-scale documents or text corpora. Commonly used topic models, such as LDA^38^ and nonnegative matrix factorization^39^, employ a bag-of-words approach, modeling documents as word frequency vectors, which only capture a collection of vocabulary^40^. By contrast, BERTopic^41^ has achieved promising results in generating contextual and sentence vector representations^42^. Therefore, in this paper, we chose BERTopic for conducting topic modeling.

#### Thematic analysis of positive sentiment

**Figure 7:**
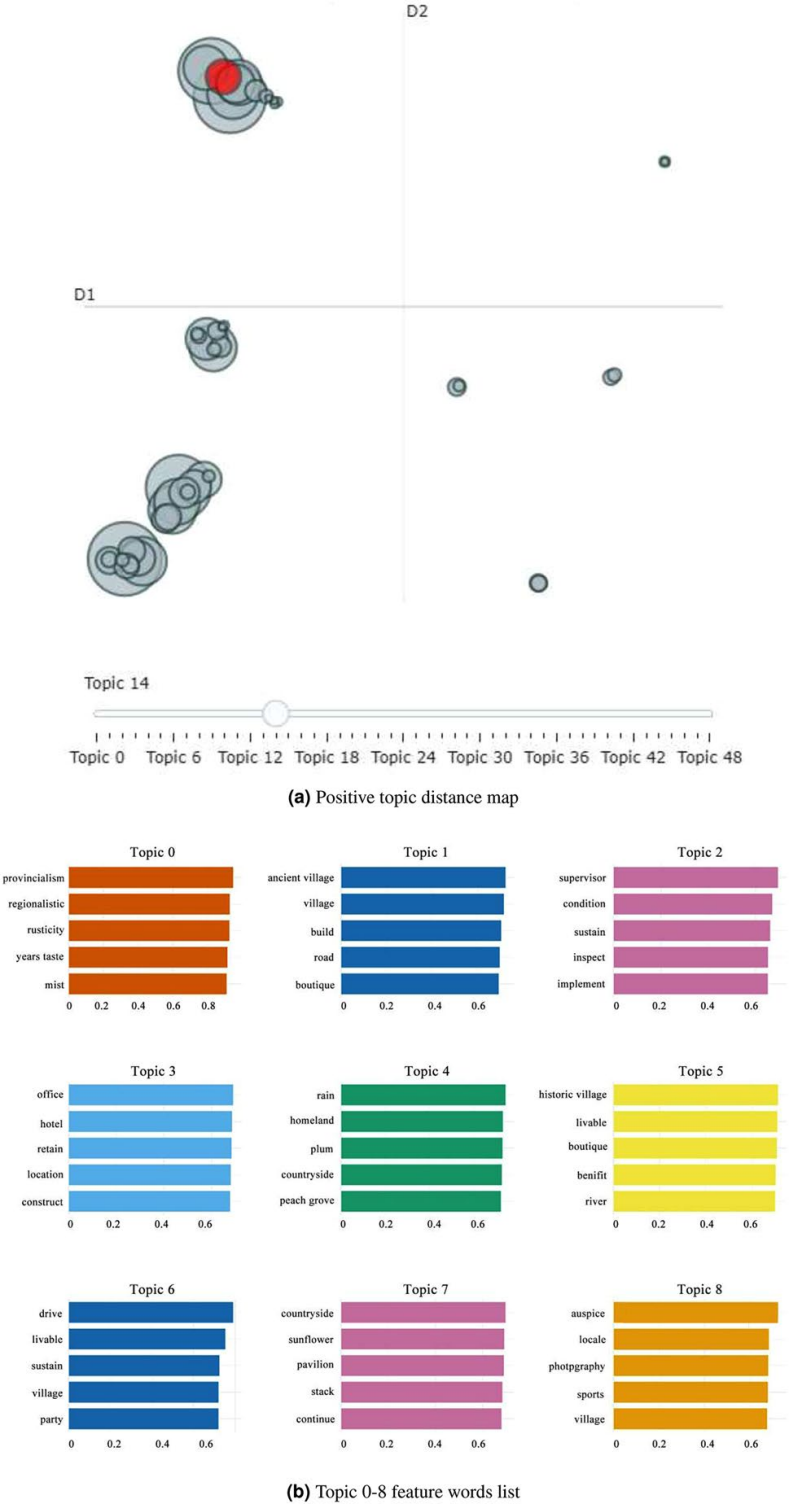
Display of positive topic.

We conducted BERTopic modeling on Weibo posts expressing positive emotions. As illustrated in Fig. 7, the automatic classification by the BERTopic model has initially identified 49 distinct topics. Each circle in the diagram corresponds to a respective topic, and a higher degree of overlap indicates greater similarity. Many topics exhibited high levels of similarity. Consequently, we implemented an automatic reduction process until the similarity score between all clusters did not exceed 0.915 (this threshold was proposed by Devlin). This method led to a reduction of our topics to 11. Subsequently, we conducted another round of model training to eliminate noise and irrelevant observations. Ultimately, our topic clustering yielded seven results, with a selection of theme words displayed in Table 1.

**Table 1:** Characteristic words of positive attitude.

Topic	Title	Feature words
0	Policies and regulations	Common prosperity, cultural city, green mountains, demonstration zone, strict control, sustainable, livable, Party branch, supervision, implementation
1	Rustic scenery	Doorstep, village, village jurisdiction, memorial hall, ancient village, beautiful, lawn lights, beautiful, model village, watchtower
2	Planning and design	Rural planning, blue bricks, talent, landscape planning, landscape architecture, industry, bright, cultural hometown, original author, artist
3	Local memory	Local flavor, treasure land, environment, landscape, New Year flavor, childhood, countryside, river, sunflower
4	Rural tourism	Beautiful, Yunnan, tourism, local literature, love, clouds, hometown, living, aged care, family
5	Plant cultivation	Seedlings, beauty plum, elm plum, red plum, clumping, survival rate, planting, tree species, Taihu stone, design institute
6	Environmental build	Layout, Xi ’an, floor space, hotel, music, fit, replacement, professional, information, matching

**Topic 0** reveals the Chinese public’s attention and response to relevant policies and regulations. Since the 21st century, the significance of rural development has gradually increased among the Chinese public. The 19th National Congress of the Communist Party of China proposed the rural revitalization strategy, with rural landscape construction becoming a core component of this strategy. The Communist Party of China and the Chinese public have actively engaged in various levels of promoting rural landscape development.

The overall content of **Topic 1** is a positive evaluation of rural scenery. “Breathtakingly beautiful” and “exquisite” directly describe the public’s pleasure when observing rural beauty.

**Topic 2** indicates China’s emphasis on talent cultivation for rural landscape planning and design. The rapid development of disciplines such as landscape architecture has deepened public understanding and research on rural landscapes. China places significant importance on the field of rural landscape planning and design to explore exceptional rural landscape planning solutions in the future.

**Topic 3** indicates the Chinese public’s pursuit of local cultural elements. The original data reveal that discussions about rural landscapes with distinctive local characteristics receive the highest engagement. People enjoy finding a sense of comfort and belonging in such environments.

The central idea of **Topic 4** is rural tourism. In this post-pandemic era, China’s rural tourism industry has experienced rapid growth. The Chinese public actively participates in rural tourism, and some individuals even consider retiring and living in rural areas.

**Topic 5** concerns local nursery planting. One of the main concerns for professionals involved in rural landscape design is the survival rate of nursery plants during the planting process.

**Topic 6** involves discussions among the Chinese public about rural environmental development, indicating a considerable focus on aspects of human habitat, such as “hotels,” during the process of rural tourism.

#### Thematic analysis of negative sentiment

**Figure 8:**
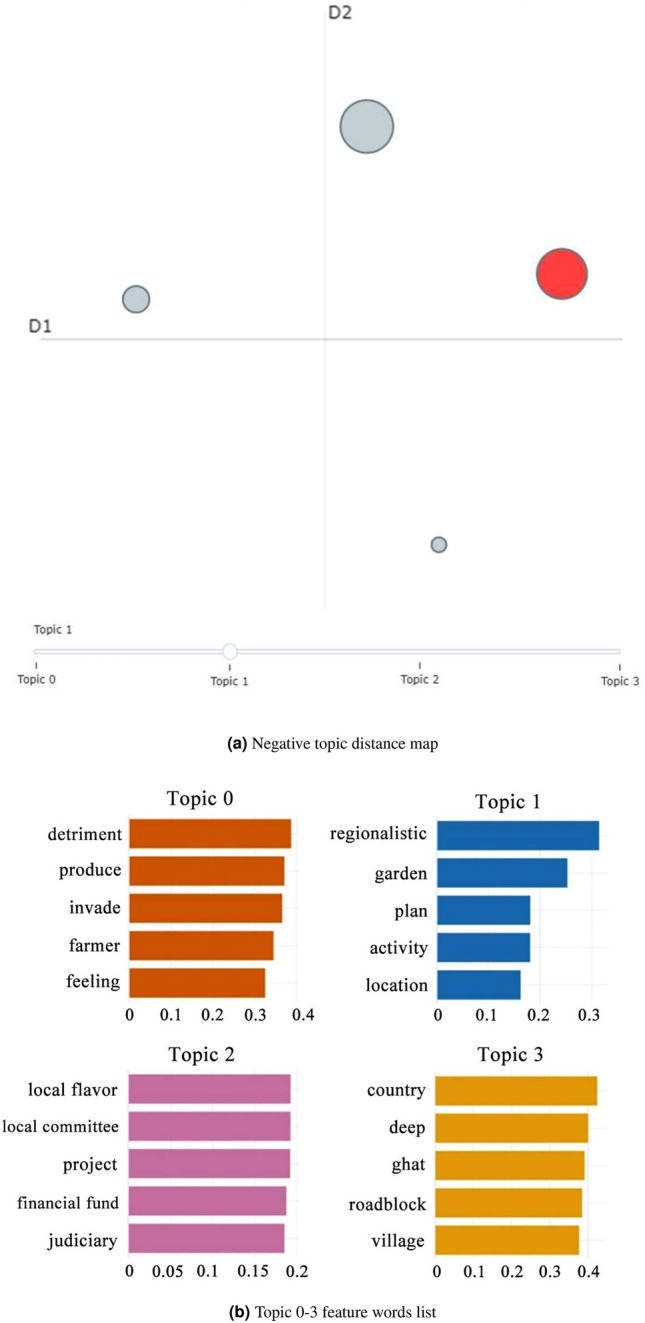
Display of negative topic.

We applied BERTopic modeling to the remaining Weibo posts expressing negative emotions to explore the potential sources of negativity that might hinder the progress of rural landscape development. As depicted in Fig. 8, BERTopic segmented the negative emotion posts into four main themes, with a selection of theme words displayed in Table 2 .

**Table 2:** Characteristic words of negative attitude.

Topic	Title	Feature words
0	Concerns of indigenous residents	Detriment, produce, invade, farmer, feeling, agriculture, crops, harvest, rural, invasion
1	Loss of local cultural elements	Regionalistic, garden, plan, activity, location, development, landscape, design, urban planning, regional development
2	Financial investment	Local flavor, local committee, project, financial fund, judiciary, community, funding, municipal, governance, budget
3	Development challenges	Country, deep, ghat, roadblock, village, rural area, barrier, natural landscape, travel, accessibility

**Topic 0** indicates that the rural landscape development has exerted a certain influence on the living spaces of indigenous residents. Rural areas serve as the primary activity sites for indigenous inhabitants. However, in the process of new rural construction and renovation, the living spaces of indigenous residents will be compressed, resulting to a sense of intrusion and crisis.

**Topic 1** addresses the incorporation of local cultural elements in landscape planning and design. To cater to modern aesthetic preferences, many designers and builders tend to adopt contemporary styles in landscape construction. Unfortunately, this often overlooks the original cultural context of rural areas, causing distress and concern among indigenous residents.

**Topic 2** highlights the lack of coordination between financial investments at the management level and the development of rural landscape planning and construction. Locations affected by this issue are often abandoned or left inactive, which not only affects the local economy but also leads to environmental degradation and wastage of resources.

**Topic 3** addresses the practical challenges encountered during rural landscape development. Many aesthetically valuable rural sceneries face difficulties in accessibility or development due to the limitations of their natural surroundings. Such challenges result in these locations being overshadowed or encountering development obstacles, causing concern among stakeholders.

### Time series analysis

**Figure 9:**
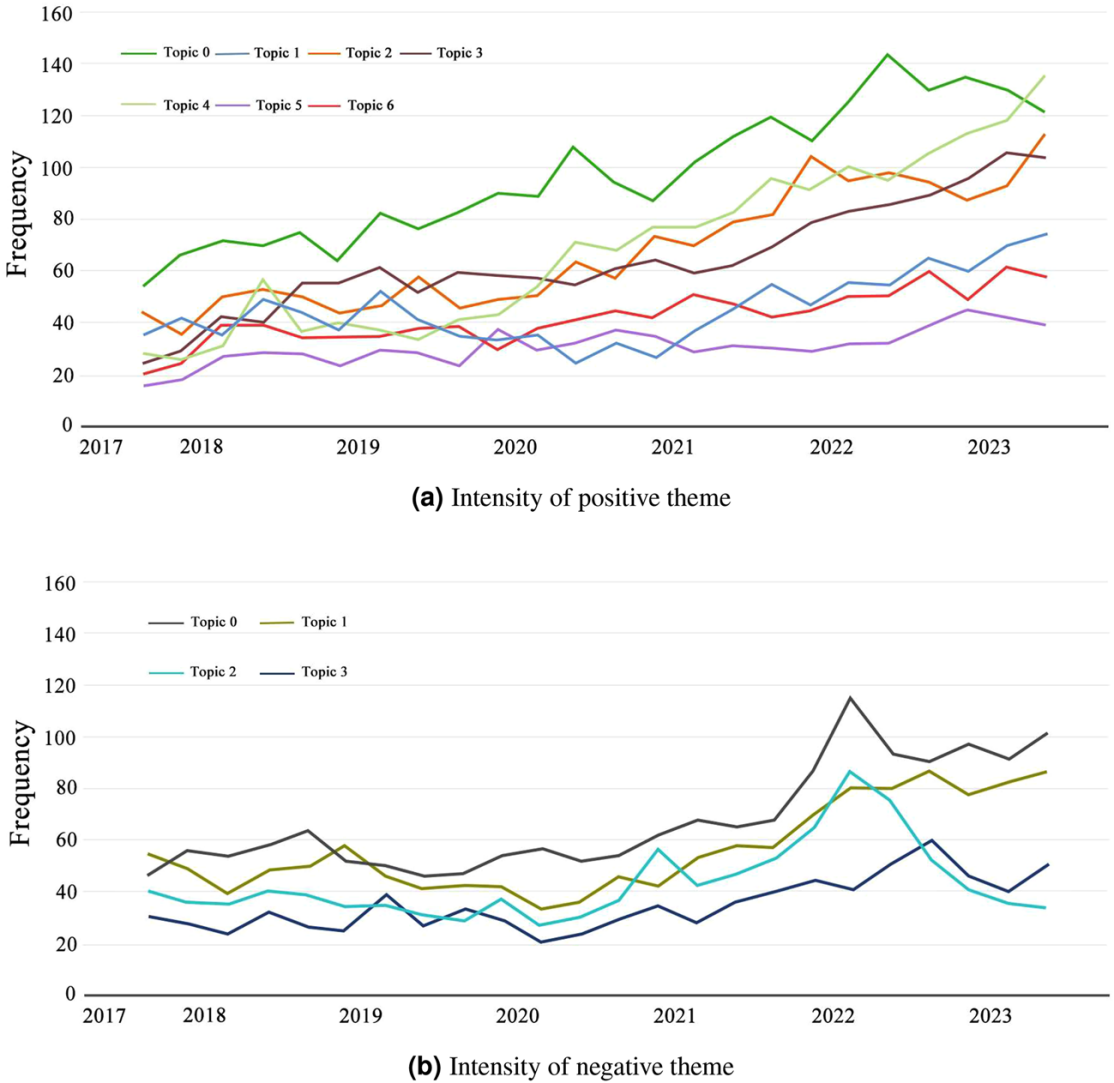
Theme intensity change trend.

The primary objective of time series analysis is to unveil patterns and regularities within data sequences for the purposes of prediction, interpretation, and decision- making. In this study, each quarter was treated as a time window for the calculation of topic intensities across seven positive and four negative themes. The results are illustrated in Fig. 9.

#### Time series analysis of positive themes

Figure 9a displays the variation in intensities of positive themes. The majority of themes exhibits a trend of increasing intensity over the years. Particularly, **Topic 4** (Rural tourism) and **Topic 2** (Talent cultivation) demonstrate the most significant growth, indicating the popularity of rural tourism and the significant attention given to talent cultivation in the field of rural planning. The intensity of **Topic 0** (Policies and regulations) fluctuates notably over time, further confirming the influence of policies on public attention and focus. We predict that over the coming period, the intensity of most positive themes will continue to rise. Rural tourism holds substantial development potential. The integration of local cultural elements remains a focal point for public exploration of rural landscapes. Chinese education will continue to delve into academic research and talent cultivation related to rural landscapes.

#### Time series analysis of negative themes

Figure 9b illustrates the variations in the intensity of negative themes. **Topic 2** exhibits a decreasing trend, indicating that with the implementation of the rural revitalization strategy and the rise of rural tourism, the willingness of the Chinese government and various industries to invest in rural landscape construction is becoming more evident. **Topic 0** , **Topic 1** , and **Topic 3** all show increasing trends. The most noticeable change is observed in **Topic 0**, revealing that the increasing rural construction activities have brought more troubles and concerns to indigenous residents. Next is **Topic 1**, indicating that a large number of rural landscape construction projects still struggle to integrate local cultural elements. In the future, due to the growing number of rural landscape construction activities, the Chinese public may exhibit more negative emotions. However, as relevant issues are gradually addressed, the sense of concern is likely to diminish over time.

## Discussion

The abovementioned study is based on the Sina Weibo platform, investigating the level of attention, emotional inclination, and focus of the Chinese public on rural landscapes. Over time, the public’s attention to rural landscapes has increased in cognition and action. The majority of the public holds a positive attitude toward rural landscapes, believing that the development of rural scenery enhances the overall wellbeing index of residents. However, a small portion of the public expresses negative emotions from different perspectives.

The number of posts shows an increasing trend year by year. At present, the grand goal of comprehensively building a moderately prosperous society in China is continuously advancing. The living standards and quality of life of the Chinese public have greatly improved. Meanwhile, rural tourism, characterized by the opportunity to experience the rural countryside and its natural beauty, provides people with a place to relax physically and mentally. The public demand for rural tourism has contributed to an increase in their attention to rural landscapes. In the coming period, we predict that the number of posts related to rural landscapes will continue to show a growing trend, partly due to the aforementioned public demand and the era of information technology^43^. Many Weibo posts exhibit the “celebrity effect,” where considerable knowledge, news, and commentaries are related to topics involving idols^44^. This era provides an opportunity for rural landscape development. When promoting and publicizing rural landscapes, leveraging celebrity and influencer effects can enhance promotional efforts. Fans are more likely to emotionally support their posts, and the influence can be expanded through professional marketing strategies in the media.

Due to regional differences, attention to rural landscape is different. In terms of the spatial distribution characteristics of post volume, the southeastern coastal regions have a higher number of posts, which is closely related to the local economic conditions and population base. By contrast, the central and western regions have lower levels of participation in rural landscape discussions. The northwestern region boasts abundant and diverse tourism resources, where natural and cultural landscapes complement each other^45^. Forests cover approximately 55% of the northwestern region’s land area nationwide, encompassing various landforms and a rich array of flora and fauna resources. The local natural landscapes are diverse, including features such as highland snow-capped mountains, waterfall streams, and forested grasslands. Simultaneously, the western region is a melting pot of various ethnic groups, hosting numerous projects with strong ethnic characteristics^46^. According to the latest statistics from China’s Digital Museum of Intangible Cultural Heritage, the western ethnic areas have 10 projects with distinct ethnic characteristics, successfully listed in UNESCO’s “Representative List of the Intangible Cultural Heritage of Humanity.” These natural environments and ethnic features can provide favorable prerequisites for rural landscape development. By tapping into and exploring the resources and cultures of the northwestern region, rural landscape development, as well as various aspects of local development in the region, can be promoted.

Policy is an important factor that guides rural landscape development. From the temporal analysis chart of post volume, a certain degree of growth in interest can be observed before and after the release of relevant national policies. In addition, discussions related to national policies in positive sentiments account for 37.9% of the overall scale. Both factors indicate that policies have played a decisive role in influencing the public’s attention to and emotions about rural landscapes. In 2015, 193 United Nations member states signed the “2030 Agenda for Sustainable Development,” jointly committing to achieving sustainable development by 2030^47^. Many goals in this agenda involve the development of rural areas, encouraging the protection of rural landscapes and promoting their sustainable development. Meanwhile, the Chinese government has issued numerous policies concerning rural landscapes, gradually influencing public perceptions. Through updates and improvements to relevant regulations, the Chinese government explicitly introduced the Rural Revitalization Strategy in October 2017. However, in comparison to foreign counterparts, China, having started relatively late, still has significant room for improvement^48^. Policymakers should establish a more detailed and comprehensive framework for rural landscape, ensuring the comprehensive development of mandatory, incentive, and supportive policies from the national to local levels. Moreover, the government should coordinate efforts at the grassroots level to promote public emotional identification. Through a combination of “government regulation” and “self-regulation,” rural landscape development can be advanced, and sustainable development goals can be achieved sooner.

The thematic content of positive emotions reflects the hot spots of public concern. In terms of rural landscape forms that capture the public’s interest, ancient villages with special significance and rural landscapes rich in local characteristics are highly favored by the public. Yu et al.’s research^49^ indicated that the satisfaction of tourists’ nostalgic emotions leads to a sense of happiness among visitors, resulting in a positive attitude toward the tourist destination and influencing recreational behavior intentions^50^. Moreover, when tourists’ nostalgic emotions are fulfilled, their willingness to revisit and their future loyalty increase. The stronger the sense of nostalgia in rural tourism is, the greater the likelihood of revisiting in person and recommending to others in the future will be. Research on nostalgic marketing and development strategies holds practical significance for tourist destinations. Scholars can segment different nostalgic groups, comprehensively understand their nostalgic motivations and preferences, and then construct rural landscape types and develop tourism products accordingly. By doing so, tourists are allowed to fully express their nostalgic emotions, thereby enhancing the value of tourist destinations and ultimately achieving rural revitalization. Furthermore, the discipline of landscape architecture has gained depth and responsibility in line with the progress of the times, playing an irreplaceable role in rural revitalization strategies^51^. Hence, a strong emphasis should be placed at the national level on nurturing professionals in related fields. Relevant scholars should master fundamental knowledge and methodologies, and take the initiative in undertaking tasks related to rural planning, design, and implementation. Establishing relevant associations, conferences, and regular exhibitions focused on rural landscapes can be encouraged, providing a platform for the public to understand and become familiar with essential knowledge in this domain.

The thematic content of negative emotions reflects the concerns of indigenous people. In discussions about rural landscapes, the majority of the public expressing negative emotions are the indigenous residents. Many rural landscape designs have overly disrupted the lives of indigenous inhabitants, leading to hostility toward external tourists among certain indigenous residents^52^. With the completion of a multitude of landscapes, the routines of rural indigenous inhabitants have been compelled to change^53^. When designers work on rural landscape projects, they must adhere to a people-centric design philosophy, focusing on the pivotal role of indigenous inhabitants. This adherence is crucial for preserving the traditional layout of living spaces for these inhabitants. Another approach is to alter the initial concepts of rural landscape design, replacing large-scale modern designs involving extensive new construction or reconstruction with designs dominated by local geographical features. This approach ensures a strong connection between the landscape and the living environment of villagers. Thus, it not only addresses the concerns of the indigenous inhabitants but also showcases the unique charm of rural landscapes, thereby promoting rapid rural economic development.

## Conclusion

As the Chinese public’s demand for material culture grows, rural tourism is widely welcomed for its nature-oriented and relaxation-inducing characteristics. The development of a favorable rural landscape not only enhances the public’s tourism experiences but also contributes to the sustainable development of rural areas. Against the backdrop of the big data era, we utilized the Sina Weibo platform, Weibo web crawling technology, and the thematic analysis model BERTopic to collect and analyze Chinese public posts related to rural landscapes. We conducted an in-depth study of the changing trends, attention status, emotional inclinations, and areas of focus. The results indicate that the Chinese public’s attention toward rural landscapes shows a gradually increasing trend year by year. In China, different regions exhibit varying levels of attention toward rural landscapes, with the Southeastern regions exhibiting greater attention than the Northwestern regions. The majority of Chinese audiences express positive emotions toward the rural landscape development, giving higher evaluations to aspects such as national policies, landscape types, and recreational effects. A small portion holds negative attitudes, reflecting a contradiction between rural landscape development and the original spatial layout.

On the basis of our empirical results, we have summarized several specific implementation methods. First, the Chinese government should play its role in constructing a more detailed and comprehensive framework for rural landscapes and actively engage in community work to achieve sound rural landscape development. Second, designers can determine the direction of rural landscape development based on the public’s emotional inclinations, where “nostalgic emotions” have practical significance for enhancing visitor loyalty. Third, promoting and publicizing rural landscapes through the use of celebrity or influencer effects can garner emotional support from fans. Fourth, organizing regular academic conferences and public lectures can enhance public understanding and attention toward rural landscapes. Fifth, delving deep into the exploration of ethnic cultures and geographical features in the northwestern inland region can drive various developments in the northwestern rural areas. Lastly, in designing and constructing rural landscapes, the opinions and needs of indigenous residents must be fully respected and considered to avoid any disruptions to their living spaces and ways of life.

Compared to existing studies, our research introduces significant innovations in both methodology and findings. Methodologically, we have implemented the BERTopic model, an advanced text mining tool that represents a significant technological advancement in analyzing public attitudes towards rural landscapes. This method enhances the accuracy of sentiment analysis beyond traditional models and facilitates a deeper comprehension of the public’s multifaceted perspectives. In terms of research outcomes, our study unveils new perspectives on the developmental trajectories of rural landscapes in China within the context of global sustainability challenges. Furthermore, by incorporating advanced technologies into the study of rural landscapes, our research addresses gaps in the existing literature and provides a novel data foundation. Overall, our study not only advances the theoretical framework but also practically applies cutting-edge technology in a meaningful way.

However, this study also has certain limitations. For instance, Weibo users may not encompass all age groups, leading to potential data incompleteness. Our research is also confined to the Weibo platform. Therefore, future studies could consider obtaining more comprehensive data sources through different platforms or multiple dimensions. Moreover, future research could integrate sentiment analysis with studies in other fields to make contributions more aligned with public needs.

## Data Availability

The datasets generated during and/or analyzed during the current study are available from the corresponding authors on reasonable request.
